# Supplementary figures and images for: Respiration rate scales inversely with sinking speed of settling marine aggregates
Source: PLoS One. 2023 Mar 1;18(3):e0282294. doi: 10.1371/journal.pone.0282294 (PMC9977048; doi:10.1371/journal.pone.0282294)

Oxygen concentration ( $\mu\text{mol L}^{-1}$ )

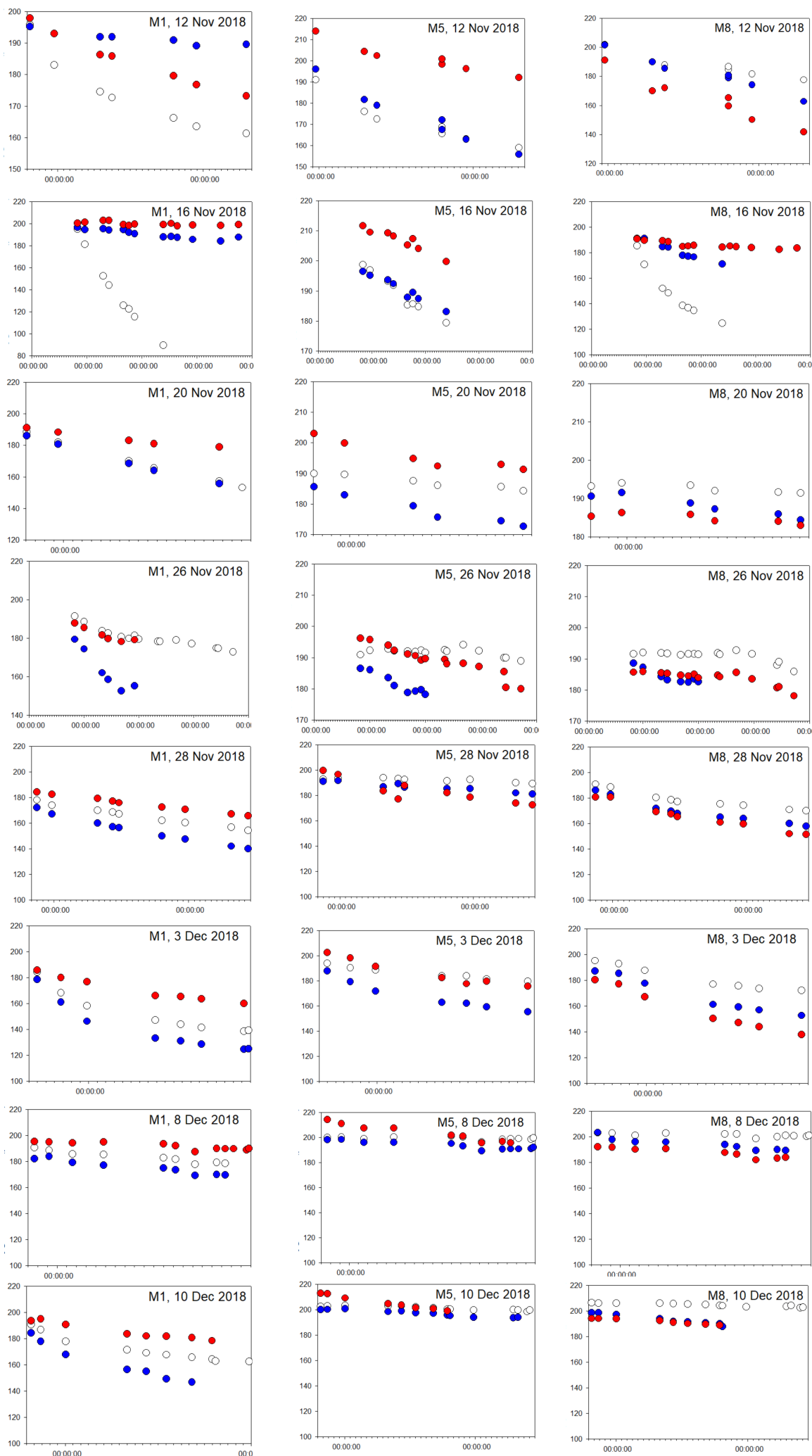

Time (clock)

Supplement: S1 Fig — The slow sinking fraction (white), mid sinking fraction (blue) and fast sinking fraction (red). The rows represent different measuring days and the columns the three mesocosms that we extracted material from (M1, M5 and M8: for the details of the setup see [16]). The x-axis represents time with 00.00.00 being midnight and each minor tick represent one hour. The oxygen respiration was calculated by linear regression from the incubation period and only significant slopes (p < 0.05) were considered. The oxygen respiration was transformed to carbon respired using a respiration quotient of 1, and normalized to the starting particulate organic carbon. (PDF) [file pone.0282294.s001.pdf]

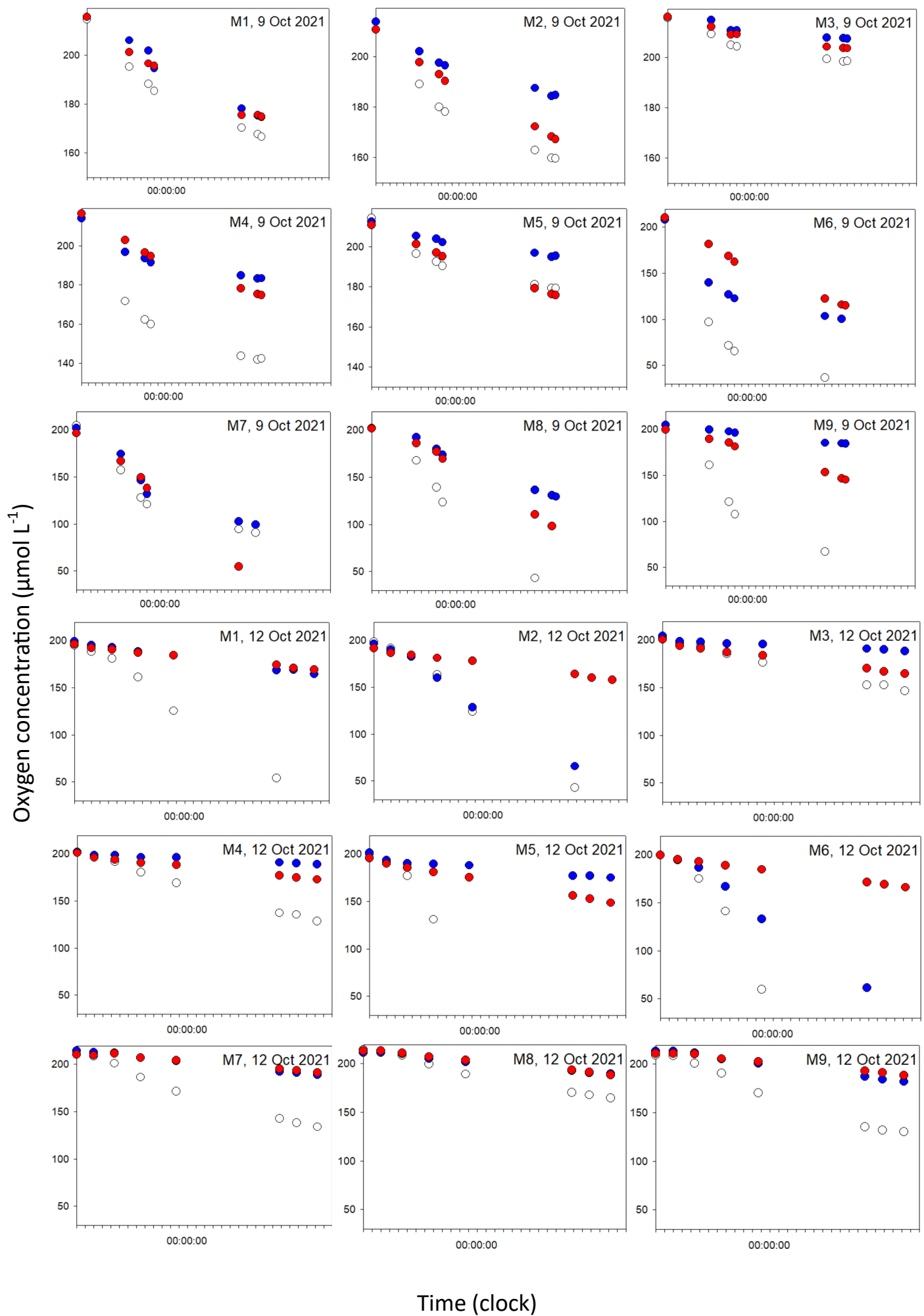

Supplement: S2 Fig — The slow sinking fraction (white), mid sinking fraction (blue) and fast sinking fraction (red). The x-axis represents time with 00.00.00 being midnight and each minor tick represent one hour. The oxygen respiration was calculated by linear regression from the incubation period and all slopes were significant (p < 0.05). The oxygen respiration was transformed to carbon respired using a respiration quotient of 1, and normalized to the starting particulate organic carbon. (PDF) [file pone.0282294.s002.pdf]
