# Supplementary material for: Respiration rate scales inversely with sinking speed of settling marine aggregates
Source: PLoS One. 2023 Mar 1;18(3):e0282294. doi: 10.1371/journal.pone.0282294 (PMC9977048; doi:10.1371/journal.pone.0282294)
Supplement: Attachment — Submitted filename: Detailed comments to review.docx [file pone.0282294.s004.docx]

**Detailed comments with author responses in red**

When submitting your revision, we need you to address these additional requirements.

1. Please ensure that your manuscript meets PLOS ONE's style requirements, including those for file naming. The PLOS ONE style templates can be found at 
<https://journals.plos.org/plosone/s/file?id=wjVg/PLOSOne_formatting_sample_main_body.pdf> and 
<https://journals.plos.org/plosone/s/file?id=ba62/PLOSOne_formatting_sample_title_authors_affiliations.pdf>.

We went through the documents and made changes to the formatting to fit the style

2. We note that the grant information you provided in the ‘Funding Information’ and ‘Financial Disclosure’ sections do not match.

When you resubmit, please ensure that you provide the correct grant numbers for the awards you received for your study in the ‘Funding Information’ section.

Corrected

3. Thank you for stating the following in the Acknowledgments Section of your manuscript: 
"We would like to thank the Oceanic Platform of the Canary Islands (PLOCAN) and the Marine Science and Technology Park (PCTM), University of Las Palmas for using their facilities. We also thank the whole KOSMOS team from GEOMAR for logistics and technical help. The mesocosm experiment in 2018 were funded by an EU ERC (Grant No. 695094) and in 2021 by EU H2020 (Grant no 869357). Additional funding came from the AQUACOSM project (EU H2020, grant 731065) and Walter and Andree de Nottbeck Foundation to KS and MV. This study utilized research infrastructure part of the Finnish Marine Research Infrastructure consortium (FINMARI)."

We note that you have provided funding information that is not currently declared in your Funding Statement. However, funding information should not appear in the Acknowledgments section or other areas of your manuscript. We will only publish funding information present in the Funding Statement section of the online submission form. 
Please remove any funding-related text from the manuscript and let us know how you would like to update your Funding Statement. Currently, your Funding Statement reads as follows: 
 "The mesocosm experiment in 2018 were funded by an EU ERC (Grant No. 695094) and in 2021 by EU H2020 (Grant no 869357). Additional funding came from the AQUACOSM project (EU H2020, grant 731065) and Walter and Andree de Nottbeck Foundation to KS and MV."

Please include your amended statements within your cover letter; we will change the online submission form on your behalf.

We removed the funding information from the Acknowledgements

4. Thank you for stating the following in your Competing Interests section:  
"NO authors have competing interests."
 
Please complete your Competing Interests on the online submission form to state any Competing Interests. If you have no competing interests, please state "The authors have declared that no competing interests exist.", as detailed online in our guide for authors at <http://journals.plos.org/plosone/s/submit-now> 
 This information should be included in your cover letter; we will change the online submission form on your behalf.

Corrected

5. Please include captions for your Supporting Information files at the end of your manuscript, and update any in-text citations to match accordingly. Please see our Supporting Information guidelines for more information: <http://journals.plos.org/plosone/s/supporting-information>.

Captions for supporting material has been added at the end of the manuscript

**Reviewer #1**:

I have read and reviewed the manuscript “Respiration rate scales inversely with sinking speed of settling marine aggregates” by Spilling et al.

The authors collect particles in-situ, separated those particles by sinking speed in a settling column, and the performed mesocosm experiments (in which oxygen loss was measured over time) to determine the relationship between size and respiration rate. At this site, on two separate years, the authors showed that fast sinking particles had lower respiration rates than slower sinking particles. However, they showed variability in the respiration rate between pools of faster sinking particles.

Particle remineralization rates and their relationship with particle size are poorly constrained in the literature, and this paper offers an important step forward. However, I had challenges understanding their exact methodology and found some of the writing confusing. I outline my concerns below.

Our original aim was to keep the text very concise, but we see that it easily leads to misunderstandings or challenges understanding what was done. To improve this, we have expanded on the methods chapter as described below.

Major comments:

Editing:
The paper needs substantial additional editing – there are numerous grammatical and other writing mistakes. I outline *some* of these in the minor comments. In general, the paper reads like the first author didn’t have enough support by the senior authors in writing. I thin k more eyes on this manuscript would help a lot.

We have carefully gone through the paper and corrected grammar and spelling errors.

Porosity:
The authors discuss porosity quite a lot, and seem to imply in places that they measure porosity. I don’t see any evidence that porosity was measured.

The reviewer is correct that porosity was not presented in the paper, and we agree that the text should be clarified. Porosity was measured in the 2018 experiment and presented in Baumann et al 2021 that presented the total (i.e. not speed separated) respiration rate. To make this clearer we have rewritten the porosity part and formulated it rather as a testable hypothesis. We have also included a new figure with examples from particles with different porosity that were sinking at different speeds.

After re-reading, the authors seemed to imply that smaller particles are more porous than larger ones. I don’t think such a relationship has been established in the literature, and indeed my understanding is that larger particles have a higher fraction of pore space than smaller ones.

There was some correlation with size, but this was not as important as porosity and we removed this sentence to avoid confusion. To our understanding, this is a field that has not receive much attention and as mentioned above we have tried to formulate this as a testable hypothesis now.

Variation in particle remineralization rate:
My understanding is that the authors measured pools of particles, rather than single particles. If I’m wrong here, or even if i’m correct, the authors should clarify. So what is up with the variability in particle remineralization rate. There is lots of variance in replicate runs?

Yes, we measured pools of particles that were differentiated into different sinking categories. This was done for the different mesocosms, so the variability is not between replicate runs but rather between different mesocosm and temporally (different days). We added more details to the data-table, and this should hopefully be more understandable now.

….

This confusing dialogue around porosity shows up in the abstract and discussion.

This has now been changed and should hopefully be clearer.

See for instance, the observation that particles with increasing diameter increase in mass at rates lower than one would expect from spherical volume alone. See the following reference:
Alldredge AL, Silver MW. Characteristics, dynamics and significance of marine snow. Progress in Oceanography. 1988;20(1):41–82.
Guidi L, Jackson GA, Stemmann L, Miquel JC, Picheral M, Gorsky G. Relationship between particle size distribution and flux in the mesopelagic zone. Deep Sea Research Part I: Oceanographic Research Papers. 2008 Oct;55(10):1364–74.

Thank you for the references

Abstract
Line 16: Sentence is not grammatical as written.
Its -> Their
corrected
Line 18: at -> in
corrected
Line 21 0.12/day (add standard deviation)
Added

Line 22: more variable: State the mean and standard deviation
added
Line 22 Slower sinking particles tened to have a higher porosity: I don’t see where porosity was measured in this paper.
reformulated
Lines 21 and 22, If the data are really non-normal consider instead reporting median and median adjusted deviation (and maybe range). This approach would be more congruent with figure 1.

We kept the mean for comparison to most other published data but included the standard deviation

22: Smaller particles should have a higher surface aria to volume ratio, even if porosity is held constant. Right? Can the authors be clear that they have deconvolved porosity and size to surface area scaling better here?

True, and a good point. The text regarding porosity was rewritten as mentioned above.

Abstract: These processes tend to be location dependent. Please disclose your sampling location in the abstract.

Added:

“Here we collected aggregates from sediment traps mounted underneath mesocosms on two occasions. These were moored off Gran Canaria, Spain (~ 27.9 N; 15.4 E) in a coastal, sub-tropical and oligotrophic ecosystem.”

Figures and Tables:

Table 1. Could we ge ta plain language dexcriptionof Diff of Ranks and Q?

Added a short sentence but explaining e.g. the Q statistics would be out of scope here, it is equivalent to the T statistic in a t-test or F statistic in a traditional ANOVA.

“The difference of rank means between groups (Diff of Ranks), the Q test statistic (Q) and the p-value (P).”

Fig 1. What do the circular dots represent? Range?

No these are data points outside the 10-90 percentile. This was added to the figure legend.

Supplementary Excel File –
What do the rows represent? My first guess is that each cel is a particle of different sinking speed and its respiration. But then I notice that there are ND columns scattered about in a sort of counterintuitive way. A longer description is clearly in order.

Yes, you are correct that each row represents one sample divided up into different sinking fractions. The no data (ND) came quite a few times during the 2018 experiment and was due to no detectible respiration. The reason for the non-detectable respiration during the incubation period was too little material in the fraction (or too short incubation period for the material added) and this was the primary reason for changing the sinking fractions slightly in the 2021 experiment to have more even distribution of material.

We have added this information to the table legend.

Also what happened when the authors report ND? Do remineralizatio detected? Sample dropped on the floor? Something else?

As explained above, the reason was too little material to detect any respiration using the incubation period and method we used. It does not imply no respiration took place, rather that we would have needed a more sensitive method or longer incubation period to be able to measure it.

Introduction
Line 38 – “whereas” implies a contradiction. Ballasting affecting sinking rates is not contradictory with respiration rate of 0.1/day

True, we changed this to

“Experimental studies using phytoplankton cultures in roller tanks have indicated that the content of ballasting minerals affects sinking speed with respiration rate independent of this at approximately 0.1 d^-1^”

Introduction: Several papers suggest that ballast minerals protect organic matter from breakdown. I recommend considering, and perhaps citing the following papers

Armstrong RA, Lee C, Hedges JI, Honjo S, Wakeham SG. A new, mechanistic model for organic carbon fluxes in the ocean based on the quantitative association of POC with ballast minerals. Deep Sea Research Part II: Topical Studies in Oceanography. 2001;49(1):219–36.
Lowenstam HA, Weiner S. On Biomineralization. Oxford University Press; 1989.
Mayer LM. Surface area control of organic carbon accumulation in continental shelf sediments. Geochimica et Cosmochimica Acta. 1994 Feb 1;58(4):1271–84.

Good point. We added some more info with the references to the introduction

Methods

Please disclose latitude, longitude of samples collected. and dates of the study.

Done

Describe the oceanography of this site for those less familiar. Such a description could go in the methods or introduction. Is it coastal? Open ocean, how eutrophic/oligotrophic, etc.

We added some information about the general conditions of the place.

I had to read the first paragraph of this methods section about three times to try to figure out what is happening. I think some additional clarity would help . In particular, as someone unfamiliar with these mesocosms, It wasn’t immediately clear to me what a sediment trap attached to a mesocosm is exactly.
In 2018 there are large KOSMOS mesocosms. In 2021 there are smaller mesocosm bags. Both setups have built in, conical, sediment traps on the bottom. Particles are collected form those traps.

Yes, they are similar with conical sediment traps in the bottom. To make it easier to understand we have included a new overview schematic of the mesocosm bags.

A visual schematic overviewing the study design would really help the reader to parse what you did.

Yes, we added this as a new Fig 1

Following my comment on the supplementary table, I don’t understand how replicates happen in this experiment and how the size fractions relate to each other.

We understand that the data presentation is unclear, and we have modified the data table. It is described in the materials and methods, but hopefully now the table is more self-explanatory. In short, they are not replicates, rather samples from different mesocosms and times.

Line 51: “attached to mesocosms” – I think the authors mean, located inside of the tanks of a mesocosm experiment, which will be summarised elsewhere?

No they were attached to the bottom of the mesocosm bags, we included a schematic that hopefully makes it clearer.

Line 54 – 15 m long. What does long mean here? Deep, or is there a length and width and depth. In which case what is the width and depth?

Yes, we mean deep here and have added the dimensions now

Line 66 68. “The settling time was seven minutes,” fine. How does this have to do with dividing up the particles by folding the tube. This seems like a sentence with two totally unrelated ideas in it.

Ok, yes, this sentence was a bit confusing. We restructured it to:

The settling time was 7 minutes, after which the tube was folded in two locations to divide the settling material into suspended, slow and fast sinking particles.

Line 69: “Not well distributed” - what does that mean? There wasn’t enough sediment to detect a respiration signal in some fractions?

Yes, this is correct. We tried to modify this to make it clearer.

It looks like the authors redefine slow, medium and fast sinkiing throughout the experiment and then merge these pools in their analysis. They should discuss what such an approach might do to their results.

We added something about this in connection with García-Martín et al (2021) coming to the same conclusion as us but also using different sinking speed fractious.

“Our division into different sinking speed fractions was different between the years, which could possibly have shifted the results slightly. However, both years fast sinking particles had lower respiration rate than slow sinking particles, strengthening the conclusion that sinking speed affects respiration rates. “

Line 82: Are the authors normalizing to carbon on the GFF, or normalizing to the sum of carbon on the GFF at the end of the experiment? This seems like too small of a number, because that ignores the carbon that was actually respired right? It seems to me like one would want to estimate the starting organic carbon concentration by summing the GFF measurements with an estimate of total respired carbon, right?

Yes, you are right, this is what we did, and we tried to make this clearer in the methods section

“The start POC was calculated by adding the calculated carbon respired to the measured POC, and the respiration rate d^-1^ was calculated by dividing respired carbon L^-1^ with the start POC concentration (L^-1^).”

Results and discussion

It is my impression that the first paragraph of this section is results and the rest is discussion. Why not split it into a results section and a discussion section?

You are right but with the new figure with example aggregates we decided to keep the combined version as it is a relatively short paper.

The authors collected particles from high nutrient and low nutrient mesocosms. But they dont’ seem to compare the results from the two different environments. Am I missing something, or did this variability just get ignored?

We did look for correlations with other variables like Chl a, but this was not conclusive, and we decided not to focus on this. There are other papers published (Special issue in Frontiers in Marine Science for the 2018 experiment) or in preparation where the nutrient concentration and general development inside the mesocosm bags are presented and discussed.

Line 89: Bold the section heading

Done

Line 96: I think you should cite figure 1 here, right?
Added

Line 93-94: No difference -> No difference in respiration rate.

Yes, added this information.

Line 105: Define slow, medium speed and fast particles in the text. Are they the same here as they are for reference 6?

No, they used suspended and <24 m d-1 as slow and >24 m d-1 as the fast-sinking fraction. We added this information to the text.

Line 119: Porosity – did you measure porosity in this site, or just size.

Yes, porosity was characterized by image analysis of a FlowCam. The details can be found in the reference, but we also now added a figure showing examples of aggregates with different porosity.

120: Sower sinking particles tended to have a higher porosity – I don’t think you measured this. If you are stating something is known, this need s citation.

Ok, this is the same reference as the sentence right before, but we now deleted this sentence as described above.

My experience is the opposite, that large particles have higher porosity.

Interesting, the connection between community composition, aggregate formation, physical form of aggregates and how this affects sinking should be studied more in detail.

Discussion overall: I’d like a better picture of why there is so much variability in the sinking speed measurements of the smaller particles.

A good point and one that we would like to answer, but we do not have any indication of what cause the higher variability. This is also why we identify this as an open question that needs further investigation.

Reviewer #2:

Review of “Respiration rate scales inversely with sinking speed of settling marine aggregates» by Spilling et al.

Comments to Authors:

This manuscript provide interesting new aspects about how respiration rates of prokaryotes attached to sinking particles are correlated with the sinking speed of the particles. The authors convincingly address their main message in a short really clear paper. I appreciate this.

I only have a few major concerns explained below and which could hopefully be addressed easily (mostly in material and method and in discussion).

Major comments:
1)The authors refer along the paper to “respiration rates of sinking particles”, e.g. L19-20 of the abstract among other. I guess you refer to respiration rates of prokaryotes attached to sinking particles or is it to associated zooplankton ? In my opinion, the message would benefit from a precision.

Yes, you are right, and we changed to this be more precise:

“Here we collected settling aggregates and determined the respiration rates of organisms (mainly heterotrophic prokaryotes) attached to aggregates sinking at different velocities. “

You are right that this is likely only prokaryotic respiration but we did not quantify the community so kept the work ‘organism’ here not to rule out any eukaryotes present.

2) Line 48, Despite a good definition and a synthetic review of what is known, I would appreciate one or two sentences more at the end of introduction convincing about why it is crucial to correlate sinking speed and respiration rates?

Good point we added these two sentences:

“The marine export of carbon to the deep ocean is a major global carbon sink, and much effort has been undertaken to quantify sinking speed of marine aggregates, which can sink 0 to 1500 m d^-1^ [11-13]. If the respiration rate scales with the sinking speed of settling aggregates, this relationship could be used to refine biogeochemical models to better quantify the biological carbon pump.”

3) Line 55 to 58 are unclear for me. I don’t get what is the “untreated control” and how adds of nutrients are done and why there. I thought it was a matter of sinking speed and respiration only?

There were more units in 2018 with a total of 9 mesocosms. The full details of the experimental setup can be found in the given reference: Bauman et al 2021. The primary measurements during both 2018 and 2021 was characterizing sinking speed of individual particles with a FlowCam. The division into different sinking speed fractions was something we did on top of the regular sampling during both experiments. Due to time constraints we were not able to do the full set of sinking speed divisions in 2018, but rather following three of the mesocosms over time. In 2021, we were able to sample all of the 9 mesocosms but only during two sampling days towards the end of the experiment once we had the routines to do the primary measurements.

We have added more details to the methods chapter, and it is hopefully more understandable now.

4) Line 67 to 73 : why sinking speed are divided differently between both experiments ? On my point of view it is not crucial for your message however it raises questions. Is that because the decantation times are not the same? Or the height of decantation ? I am just curious.

This was a point raised by Reviewer #1 as well. The problem was the distribution of the organic matter. In 2018 there were some fractions where the respiration was below the detection limit and we did not get any data. In 2021 we did initial tests first to get a more even distribution of the material, and we tried to better explain this in the text:

“There were some occasions when there was not enough sediment material in one or more sinking speed fractions to be able to measure respiration during the incubation period (below detection limit). To prevent this, we did initial tests in 2021 and changed the division to <50 m d^-1^, 50-130 m d^-1^ and >130 m d^-1^ for slow, medium speed and fast sinking, respectively. This provided a more even distribution of sinking material between the sinking speed fractions and ensured a measurable decrease in O_2_ concentration during the incubation period in all sinking speed fractions. “

5) My major concern regarding this paper is about the method to determine respiration rates (line 77 to 86). The manuscript would clearly benefits from additional descriptions here.
I saw that you did blank controls. Is it one bottle dedicated to blank that you used after to remove “blank signal” from the other incubation bottles ? Or did you perform blank in each of the bottle used prior (or after) the experiment ? If I well understand what is written, this was the first option. In that case, did you intercalibrate all bottles by doing 100% - 0% on all sensitive membranes ? In my opinion this is mandatory. If sensitive membranes are still stick inside bottles, it would be easy to check and add it.

Yes, it was the first option, i.e. blank was done in triplicates in 2018 and six bottles in 2021. Yes, each of the membranes were individually calibrated. Added text:

“All of the membranes had been calibrated using 0% and 100% air saturation of O_2_ following the protocol of the manufacturer: in short 100% by bubbling with air and 0% by addition of sodium dithionite. “

In addition, regarding data processing:
- did you verified if respiration data are well linear prior the regression? Could you add (maybe in supp data) residual distribution to check if the model fitted was the best ?
- Was the effect of temperature taken into account ?
- I am really curious about the method you choose to pick the (or one of the) linear part of the curve. This information should be described in the material and method. I would like to have in supp data the raw curve obtained.

Yes, we added more details to the methods section. So first, yes all of the bottles used had been individually calibrated against 0% and 100% O2 concentration. Second, the blanks were used to check if there was any respiration in the filtered water that was added to the sediment to fill up the bottle (completely filled with no headspace). Third, yes the measurements were temperature compensated. The thermometer was placed in a ‘dummy’ bottle that was having the same water and placed together with the measurements bottles under the assumption that all the bottles had the same temperature as they were all stored in the same place during incubations. Forth, for the linear regression we used all the measurement point during the incubation period.

We have added the raw data to the supplementary material.

6) Regarding the discussion:
- though results are well compared with respiration rates of literature, prokaryotic heterotrophic production (PHP) are related to respiration and paper comparing PHP rates of sinking speed fractions could be relevant (e.g. Church et al. 2021, Baumas et al. 2021 are examples). For instance Church et al. found a higher PHP rate for slow sinking particles than fast sinking. This could confirm your results. Baumas et al. found higher rates for suspended particles than fast sinking per volume of water but the opposite per unic cell of prokaryotes.

Thank you for the references, these were interesting, and we have included them in both the introduction and added a new section in the discussion about this. Church et al 2021 did not differentiate between sinking speeds as far as we can see, if we found the right article: Limnol Oceanogr 66: 3255-3270

Your results are convincing. The rate of respiration is inversely proportional to the sinking speed of the sinking marine aggregates. However, I am a bit frustrated at the end of the discussion. I am curious about how you would explain these inversely relation? Could it be because bacteria are more stressed by the sink when the speed is faster (e.g. pressure increasing, temperature and light decreasing..)? Is it more a matter of porosity as you mention in the introduction ?
What are the implications in term of Bacterial growth efficiency (BGE = PHP/(PHP+PR) with PR the respiration rate), on how marine aggregates are remineralized and thus on sequestration of carbon?

We were also discussing the issue of porosity and have now formulated this as a hypothesis. We have reformulated and expanded on this part, which hopefully now has a better explanation although we do not have answers to all questions e.g. what causes the variability in the slow sinking fraction. We did not measure prokaryotic production and can have consequently no direct measure of BGE, but with PHP equal, the BGE should be higher in the fast-sinking fraction.

Minor comments:
Sometimes authors refer to “aggregates” and sometimes to “particles”. In my opinion, it is better to choose one and to stay consistent (including title and abstract)

We changed this to aggregates throughout to be consistent.

Line 30-31, regarding the topic, it would be interesting and informative to add general sinking speed range with references. Here some examples among other : from 2 to 1500 m d-1 (Alldredge and Silver 1988; Armstrong et al. 2002; Trull et al. 2008). In addition, as my previous comment, I would choose between sinking velocity and speed to help being consistent.

We added the references and changed ‘velocity’ to ‘speed’ in most places.

Line 34 : I understand by this sentence that export of Carbon is related only on sinking speed or biological processes. I would add something like “among other” as additional physical, chemical or even composition of particles can act as well (all interconnected finally).

We added this according to the suggestion.

Line 52 : Could it be possible to add a “see after” when referring to respiration rates few paragraphs above its description? I was a bit lost at first but it may be only me…

Good point, we added: “(see below)” .

Line 60 : “sediment was collected” I guess you mean sinking particles which have sedimented and was collected by traps ? Or is it real sediment in the floor of mesocosms? (same question for Line 62)

Yes, it was the material collected from the sediment traps. We clarified this in the text.

Line 60 to 61 : “day 29 and 31” Why did you choose these 2 time points ? I guess it is no so important but I am just curious.

Our primary measurements during the experiment were different (measuring sinking speed of individual aggregates), and the results presented here were add-on measurements that were done during both experiments but unfortunately not for the full set. So the time points were in that sense arbitrary, but in 2021 late in the experiment when the normal measurements had become routine and there was some extra time to do these sinking speed fractionations.

I have a question about the rates in “per day”. As it is normalized by POC concentration I guess you mean “by C per day”. Is it mg ? or something else of C per day ? I am not perfectly sure about this

Oxygen decrease (respiration) was converted to C respired using a respiration quotient of 1 as explained in the methods chapter. This has then the units: µg C L-1 d-1 respired, which is divided by the particulate organic carbon concentration in µg C L-1. The µg C L-1 cancels each other out:

(µg C L-1 / µg C L-1) / d

and we are left with the rate d-1.
